# Isolation of *Microbulbifer* sp. SOL66 with High Polyhydroxyalkanoate-Degrading Activity from the Marine Environment

**DOI:** 10.3390/polym13234257

**Published:** 2021-12-04

**Authors:** Sol Lee Park, Jang Yeon Cho, Su Hyun Kim, Shashi Kant Bhatia, Ranjit Gurav, See-Hyoung Park, Kyungmoon Park, Yung-Hun Yang

**Affiliations:** 1 Department of Biological Engineering, College of Engineering, Konkuk University, Seoul 05029, Korea; shckd2020@naver.com (S.L.P.); whwkddus1123@gmail.com (J.Y.C.); gsm06136@naver.com (S.H.K.); shashibiotechhpu@gmail.com (S.K.B.); rnjtgurav@gmail.com (R.G.); 2 Institute for Ubiquitous Information Technology and Applications, Konkuk University, Seoul 05029, Korea; 3 Department of Biological and Chemical Engineering, Hongik University, Sejong 30016, Korea; shpark74@hongik.ac.kr (S.-H.P.); pkm2510@hongik.ac.kr (K.P.)

**Keywords:** poly(3-hydroxybutyrate), bioplastics, biodegradation, screening, *Microbulbifer* genus

## Abstract

Having the advantage of eco-friendly decomposition, bioplastics could be used to replace petroleum-based plastics. In particular, poly(3-hydroxybutyrate) (PHB) is one of the most commercialized bioplastics, however, necessitating the introduction of PHB-degrading bacteria for its effective disposal. In this study, *Microbulbifer* sp. SOL66 (94.18% 16S rRNA with similarity to *Microbulbifer hydrolyticus*) demonstrated the highest degradation activity among five newly screened *Microbulbifer* genus strains. *Microbulbifer* sp. SOL66 showed a rapid degradation yield, reaching 98% in 4 days, as monitored by laboratory scale, gas chromatography-mass spectrometry, scanning electron microscopy, gel permeation chromatography, and Fourier transform infrared spectroscopy. The PHB film was completely degraded within 7 days at 37 °C in the presence of 3% NaCl. When 1% xylose and 0.4% ammonium sulfate were added, the degradation activity increased by 17% and 24%, respectively. In addition, this strain showed biodegradability on pellets of poly(3-hydroxybutyrate-*co*-4-hydroxybutyrate), as confirmed by weight loss and physical property changes. We confirmed that *Microbulbifer* sp. SOL66 has a great ability to degrade PHB, and has rarely been reported to date.

## 1. Introduction

Poly(3-hydroxybutyrate) (PHB) is a well-studied bioplastic that can be produced from renewable biomass having equivalent strength and durability to petroleum-based plastics [1]. Other than its compatibility with petroleum-based plastics, it has an advantage of using low-cost substrates such as used oil or food wastes, which result in diverse length and properties of the PHA from short chain length (SCL-PHA), containing 3–5 monomer with solid texture, to medium chain length (MCL-PHA), containing 6–14 monomer with flexibility [2]. Also, with its great advantage of biodegradability, it is widely commercialized in packaging, medical devices, and hygiene disposables. With respect to biodegradation of the PHA, it can be degraded by microorganisms under unfavorable conditions of limited carbon and nitrogen sources. In this situation, the microorganism forms colonies on the surface of the polymer and hydrolyzes it by producing hydrolytic enzymes, such as PHB depolymerase and 3HB hydrolase, so that it can take up the degraded monomer as a carbon source and survive [3,4]. Only a few bacteria are known to degrade PHB, including, *Comamonas testosteroni*, *Alcaligenes faecalis*, and *Cupriavidus necator* [5,6,7]. Therefore, much effort has been made to screen and characterize PHB-degrading bacteria to broaden the biodegradation database and support commercialization of the PHB by solving its disposal issue.

The genus *Microbulbifer* consists of gram-negative gammaproteobacteria, which reside in high-salt environments with surface blebs and is rarely known for biodegradation activity in PHB. Rather, *Microbulbifer* produces pigments and antibiotics or decomposes various polysaccharides, such as alginate, cellulose, agarose, and many other complex carbohydrates, with a considerable number of strains isolated from seaweed and its waste [8,9]. Recently, *Microbulbifer hydrolyticus* sp. IRE-31T was reported to decompose low-density polyethylene [10]; however, the PHB degradation activity of the *Microbulbifer* genus has only recently been reported by our group. An increasing number of trials have been conducted to apply PHB at the industrial level, and the trials are regarded as an essential procedure to accumulate knowledge on the biodegradation of PHB.

In this study, we isolated PHB-degrading strains and selected one of them, *Microbulbifer* sp. SOL66 that showed strong biodegradation activity. We demonstrated its biodegradability by measuring the degradation yield (%) with residual PHB (mg). We also established the optimal conditions for PHB degradation with respect to temperature, salt concentration, and carbon and nitrogen source concentrations. Additionally, we conducted a physical property analysis, which showed that some changes occurred in the PHB film after biodegradation, demonstrating the effective biodegradation activity of *Microbulbifer* sp. SOL66.

## 2. Materials and Methods

### 2.1. Chemicals

All chemicals used in this study were of analytical grade. Solvents used in this study, including chloroform and dichloromethane (DCM), were obtained from Sigma-Aldrich(MA, USA). PHB pellets were obtained from Goodfellow (Huntingdon, UK), and other bioplastic pellets and N-lauroylsarcosine sodium salt (Sarkosyl NL) were purchased from Sigma-Aldrich (MA, USA). Carbon and nitrogen sources were obtained from Junsei (Tokyo, Japan).

### 2.2. 16S rRNA Sequencing and Phylogenetic Analysis

Colonies forming clear zones on the PHB plates were identified at the species level using 16S rRNA sequencing by polymerase chain reaction amplification using the primer 27F. Partial sequences were obtained by Bionics (Seoul, South Korea) and compared to those in the NCBI GenBank database (https://blast.ncbi.nlm.nih.gov/Blast.cgi, accessed on 6 August 20) using BLASTN tools. Evolutionary analysis was conducted in MEGA X (http://www.megasoftware.nte, accessed on 6 August 20) using the neighbor-joining method, and all ambiguous positions were removed for each sequence pair (pairwise deletion option). The percentage of replicate trees in which the associated taxa clustered together in the bootstrap test (2000 replicates) was determined.

### 2.3. Preparation of Bioplastic-Emulsified Medium

For the preparation of the media plates containing bioplastics (i.e., polylactic acid; PLA, polybutylene succinate; PBS, polybutylene adipate terephthalate; PBAT, polycaprolactone; PCL, poly(3-hydroxybutyrate-*co*-4-hydroxybutyrate) [P(3HB-*co*-4HB)], and poly(3-hydroxybutyrate-*co*-3-hydroxyvalerate) [P(3HB-*co*-HV)]), 0.2 g of the bioplastic pellets was dissolved in 40 mL of DCM in a 60 °C water bath for 2 h. After the bioplastics had dissolved in DCM, 2 mL of 2% Sarkosyl NL and distilled water were added and mixed thoroughly using a Vibra-Cell VCX500 (Sonics & Materials, Inc., Newtown, CT, USA) with 15 s of pulse and an amplitude of 40% for 10 min. As a result, the dissolved bioplastics in the solvent phase were uniformly emulsified in the water phase, and the mixture became an opaque emulsion [11]. After sonication, the solvent was completely evaporated in a fume hood using a stirrer at 60 °C to prevent cell damage due to the remaining solvent. Finally, marine broth (MB) and agarose were added and autoclaved [12,13].

### 2.4. PHB Degradation Assays

PHB degradation was identified by measuring the residual PHB film after the biodegradation progressed. The PHB film was prepared using the solvent cast method [14]. PHB pellets (1 g) were completely emulsified in 100 mL of chloroform for 16 h at 60 °C, and the solvent was evaporated completely in a fume hood until a plastic film was formed. It was then cut into small pieces, weighing 20 mg each, and autoclaved at 121 °C for 15 min. The sterilized PHB films were then cultured in 5 mL of liquid MB with isolated PHB-degrading bacteria in a rotary shaker at 200 rpm. After cultivation, the residual PHB films were collected, washed with distilled water, and freeze-dried for gas chromatography (GC) analysis. In addition to the liquid assay, PHB degradability was confirmed using a solid-based method. Paper discs (Toyo Roshi Kaisha, Japan) were placed in the middle of the PHB plate, and 10 μL of the culture medium was inoculated onto it. After cultivation, the radius of the clear zone formed around the paper disc was measured [15,16].

### 2.5. GC Analysis

The residual PHB was measured using GC as previously described with a slight modification [17,18,19,20]. For analysis, the culture medium was centrifuged at 10,000× *g* for 10 min to collect the residual PHB films and rinsed with deionized water to remove the cell residue attached to the film. The residual PHB films were lyophilized in Teflon-stoppered glass vials. For methanolysis of the PHB samples, 1 mL of chloroform and 1 mL of a methanol/sulfuric acid (85:15 *v/v*) mixture was added to the vials and heated at 100 °C for 2 h. The samples were then cooled at 25 °C for 10 min, and 1 mL of ice-cold water was added. Then, the samples were mixed thoroughly. The organic phase was extracted using a pipette and transferred to clean borosilicate glass tubes containing anhydrous sodium sulfate, which absorbed the remaining water, and then filtered. The samples were subsequently injected into a GC instrument (Young Lin Tech, Korea) using an HP-FFAP column (30 m × 0.32 mm × 0.25 μm); Agilent Technologies, Santa Clara, CA, USA). The split ratio was 1:10. Helium was used as the carrier gas, and the flow rate was maintained at 3.0 mL/min. A 2-μL portion of the organic phase was injected using an autosampler. The inlet temperature was maintained at 240 °C. The column oven was held at 80 °C for 5 min, heated to 220 °C at 20 °C/min, and then held at 220 °C for another 5 min. Peak detection was performed using a flame ionization detector maintained at 230 °C [14].

### 2.6. Metabolite Analysis

GC-mass spectrometry (MS) was used to detect and quantify fatty acids following a previously described method, with slight modifications. For methanolysis of the fatty acids, approximately 10 mg amounts of freeze-dried cells were weighed and placed in Teflon-stoppered glass vials. Then, 1 mL chloroform and 1 mL methanol/H_2_SO_4_ (85:15 *v/v*%) were added to the vials. Afterward, they were incubated at 100 °C for 2 h, cooled to 25 °C, and incubated on ice for 10 min. After adding 1 mL ice-cold water, the samples were thoroughly mixed by vortexing for 1 min and then centrifuged at 3521× *g*. The organic phases at the bottom were extracted using a pipette and moved to clean borosilicate glass tubes containing Na_2_SO_4_. GC-MS was performed using a Perkin Elmer Clarus 500 gas chromatograph connected to a Clarus 5Q8S mass spectrometer at 70 eV (m/z 50–550; source at 230 °C and quadruple at 150 °C) in electrospray ionization mode with an Elite 5 ms capillary column (30 m × 0.25 mm i.d. × 0.25 mm film thickness; Perkin Elmer, USA). Helium was used as the carrier gas at a flow rate of 1.0 mL/min. The inlet temperature was maintained at 300 °C. The oven temperature was programmed at an initial temperature of 150 °C for 2 min before increasing to 300 °C at a rate of 4 °C/min. The temperature was maintained for 20 min. The injection volume was 1 μL with a split ratio of 50:1. The structural assignments were based on the interpretation of the mass spectrometric fragmentation and confirmed by comparison with the retention times and fragmentation patterns of the standards and spectral data obtained from the Wiley (http://www.palisade.com, accessed on 6 August 20) and National Institute of Standards and Technology (http://www.nist.gov, accessed on 6 August 20) online libraries. Methyl heneicosanoate (10 mg/mL; 10 μL) was used as an internal standard [21].

### 2.7. Analysis of Physical Properties

Scanning electron microscopy (SEM) was performed to observe the changes on the surface of the PHB film after degradation. For SEM analysis, the residual PHB films on each day were collected by centrifugation and fixed with 2% buffered glutaraldehyde overnight at 4 °C. Glutaraldehyde was decanted after centrifugation, and the samples were washed three times with phosphate buffer to remove residual glutaraldehyde. The samples were then dehydrated using a gradually increasing ethanol concentration (50%, 70%, 95%, and 100%). Finally, different ratios of ethanol and hexamethyldisilazane (HMDS; 2:1, 1:1, 1:2 *v/v*) were used for chemical drying; 100% HMDS was used in the final step, and the mixtures of HMDS and samples were mounted on specimen stubs. The HMDS was evaporated overnight in a fume hood. The samples were then coated with gold dust at 5 mA for 120 s. Back-scattered electron images were obtained using a scanning electron microscope (TM4000Plus; Hitachi, Ltd., Tokyo, Japan) at an accelerating voltage of 5 kV [14].

The differences in the functional groups of the PHB film were detected using Fourier transform infrared spectroscopy (Nicolet 6700; Thermo Fisher Scientific, Waltham, MA, USA) in the scanning range of 4000–600 cm^−1^. The resolution was set to 4 cm^−1^, and 32 scans were recorded for each spectrum using an auto base [22,23].

Gel permeation chromatography (Youngin Chromass, Korea) was performed to determine the molecular weight and molecular mass distribution of the degraded PHB films. For sample preparation, the residual PHB films were dissolved in chloroform and stirred constantly using a thermoshaker at 60 °C for 2 h. After dissolving the PHB film, ice-cold water was added at three times the volume of the dissolved PHB solution and mixed thoroughly. Then, the precipitated solvent phase was collected, and the solvent was evaporated. Finally, it was dissolved in chloroform again, resulting in an easily dissolvable PHB sample with impurities in the sample removed. This solution was filtered through a syringe filter (Chromdisc, Korea) with a pore size of 0.2 μm to separate the dissolved PHB from the remaining insoluble cell components. A high-performance liquid chromatography (HPLC) apparatus consisting of a loop injector (Rheodyne 7725i; Sigma-Aldrich, MA, USA), an isocratic pump with dual heads (YL9112), column oven (YL9131), columns (Shodex, K-805, 8.0 I.D. × 300 mm; Shodex, K-804, 8.0 mm I.D. × 300 mm; Showa Denko K.K., Tokyo, Japan), and a refractive index detector (YL9170) was used for analysis. A total of 60 μL of the solution without air bubbles was injected. Chloroform was used as the mobile phase at a flow rate of 1 mL/min and a temperature of 35 °C. The data were analyzed using YL-Clarity software for a single YL HPLC instrument (Youngin Chromass). The molecular masses were analyzed relative to polystyrene standards ranging from 5000 to 2,000,000 g/mol [13,24,25].

### 2.8. Statistical Analysis

All data are representative of replicate experiments. Statistical significance was determined by one-way analysis of variance using MiniTab 18 software at a 95% confidence level, and statistical significance was set at *p* < 0.05.

## 3. Results and Discussion

### 3.1. Isolation of PHB-Degrading Bacteria and Selection of Microbulbifer Genus

**Figure 1:**
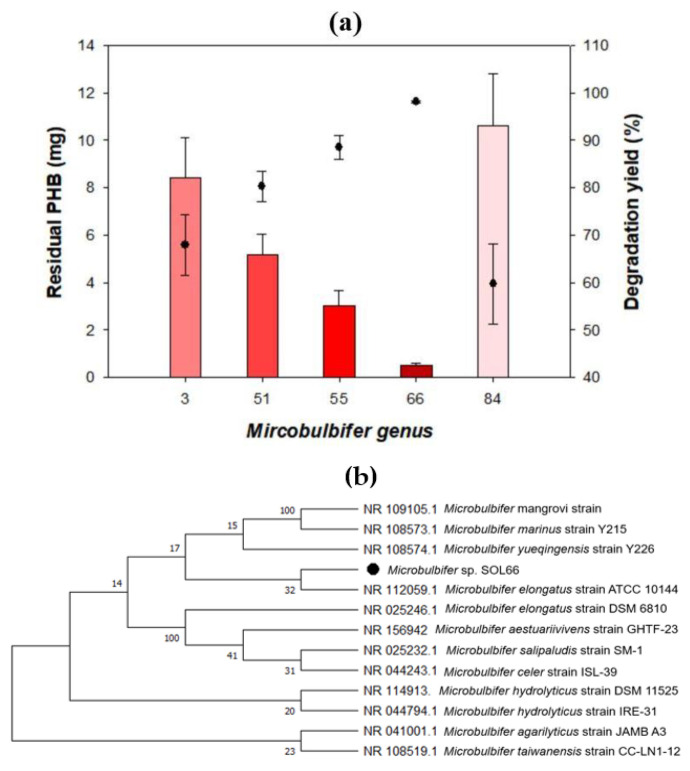

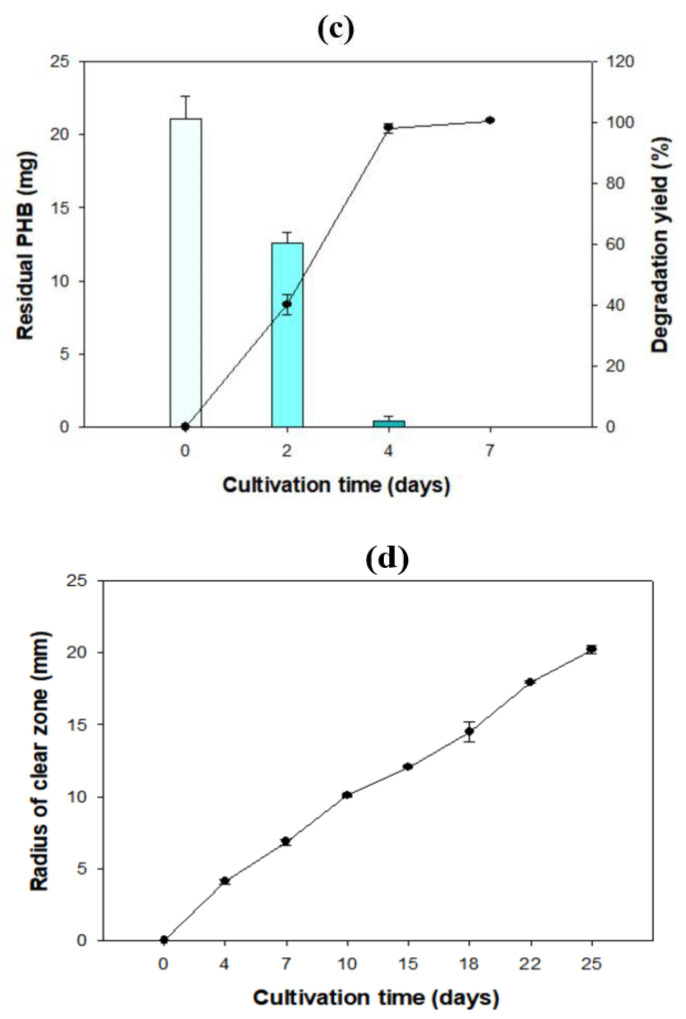
Superior poly(3-hydroxybutyrate) (PHB) degrading activity of *Microbulbifer* sp. SOL66. (**a**) The PHB degradation yields of 5 strains of *Microbulbifer* were measured using a liquid-based method. The residual PHB (mg) measured by gas chromatography is presented on the left axis, and the degradation yield (%) calculated by comparing the residual PHB (mg) to the original amount of PHB is presented on the right axis. (**b**) The phylogenetic tree is based on the sequence of the 16S rRNA. (**c**) Degradation yield (%) of *Microbulbifer* sp. SOL66 measured over 7 d. (**d**) The *Microbulbifer* sp. SOL66 cells were cultured on solid MB medium with emulsified PHB at 37 °C.

PHB-degrading bacteria were isolated from soil samples collected from Korean seashores. A considerable number of strains were identified as belonging to the *Microbulbifer* genus and showed decent biodegradability among other isolates (Table 1). Five isolates, *Microbulbifer* sp. SOL03 (95.81% 16S rRNA with similarity to *Microbulbifer taiwanensis*), *Microbulbifer* sp. SOL51 (95.15% 16S rRNA with similarity to *Microbulbifer aesturariivivens*), *Microbulbifer* sp. SOL55 (96.94% 16S rRNA with similarity to *Microbulbifer salipaludis*), *Microbulbifer* sp. SOL66 (94.18% 16S rRNA with similarity to *Microbulbifer hydrolyticus*), and *Microbulbifer* sp. SOL84 (97.01% 16S rRNA with similarity to *Microbulbifer elongatus*) formed distinct clear zones on the PHB emulsified plate only for a few days. Subsequently, they were cultured in a liquid MB with 20 mg of PHB film for 4 d at 37 °C to identify their biodegradability using a liquid-based method (Figure 1a). The residual PHB (mg) was measured by GC, and the degradation yield (%) was calculated relative to the original amount of PHB (mg).

All the bacterial strains showed a great degradation yield of more than 60% in 4 d, especially *Microbulbifer* sp. SOL66 showed a considerably high degradation yield of 98% and completely degraded the PHB film within 7 d. Given its high degradation yield, *Microbulbifer* sp. SOL66 was selected for further experiments. To infer the evolutionary relatedness of *Microbulbifer* sp. SOL66 in the genus, a phylogenetic tree was constructed based on the sequence of the 16S rRNA sequence (Figure 1b).

For a detailed study of the biodegradation activity of *Microbulbifer* sp. SOL66, liquid and solid cultures were used. For liquid cultivation, the strain was cultured with 20 mg of the PHB film in MB medium at 37 °C, and the residual PHB films (mg) were measured using GC (Figure 1c). During the first two days, there was a rapid decrease in the amount of residual PHB (mg) in a short time, reaching approximately 40% degradation yield. For the next two days, the degradation activity accelerated, and the degradation yield reached approximately 98%, which is a considerable decrease in the PHB film compared to the intact film. Finally, on day 7, the PHB film completely disappeared, and only cell debris remained. It is clear that *Microbulbifer* sp. SOL66 degraded 20 mg of the PHB film over 4 days, which showed remarkable biodegradability. In addition to the liquid culture, *Microbulbifer* sp. SOL66 cells were cultured on a solid MB medium with emulsified PHB, and the radius of the clear zone was measured (Figure 1d). The radius of the clear zone gradually increased, reaching 20 mm on day 25, and stopped growing. 

### 3.2. Optimal Conditions for Biodegradation

**Figure 2:**
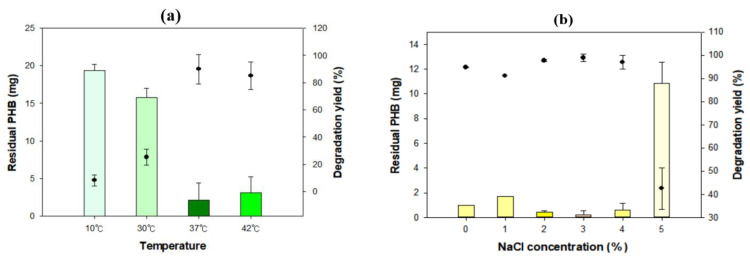

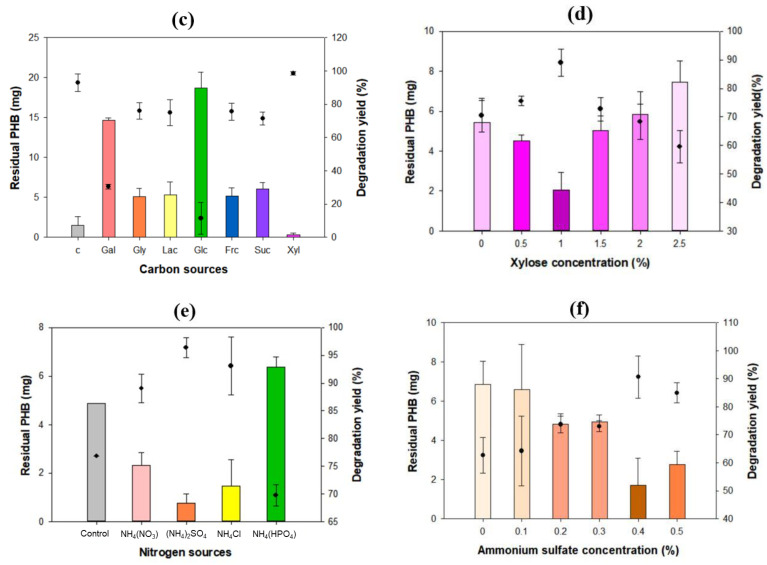
Optimal conditions for poly(3-hydroxybutyrate) (PHB) degradation (**a**) *Microbulbifer* sp. SOL66 cells were cultured at various temperatures ranging from 10 °C to 42 °C. (**b**) *Microbulbifer* sp. SOL66 cells were cultured with various concentrations of NaCl, ranging from 0% to 5%. (**c**) Various carbon sources were added, including galactose (Gal), glycerol (Gly), lactose (Lac), glucose (Glc), fructose (Frc), Sucrose (Suc), and xylose (Xyl). (**d**) Different concentrations of the xylose were added from 0% to 2.5% to determine the optimal concentration of the xylose. (**e**) Various nitrogen sources were added, including ammonium nitrate [(NH_4_)NO_3_], ammonium sulfate [(NH_4_)_2_SO_4_], ammonium chloride [(NH_4_)Cl], and ammonium dihydrogen phosphate [(NH_4_) H_2_PO_4_]. (**f**) Different concentrations of the ammonium sulfate were added from 0% to 0.5% to determine the optimal concentration of the ammonium sulfate.

To maximize the degradation activity of *Microbulbifer* sp. SOL66, the optimal conditions with respect to temperature, salt concentration, and carbon and nitrogen source addition were established. *Microbulbifer* sp. SOL66 cells were cultured with the PHB films at various temperatures ranging from 10 °C to 42 °C for 4 d, and the residual PHB (mg) was measured using GC (Figure 2a). It was observed that the PHB film did not grow or degrade at 10 °C, leaving the film almost intact. The degradation yield gradually increased as the cultivation temperature increased to 37 °C, representing the highest degradation yield of approximately 90%. However, a decreased degradation yield was shown at 42 °C, indicating that the optimal temperature for *Microbulbifer* sp. SOL66 to degrade PHB film was 37 °C. This might be related to the optimal temperature for its growth, which is also 37 °C [8]. In addition to the temperature, the optimal salt concentration for biodegradation was observed (Figure 2b). Additional NaCl was added to adjust the salt concentration to 5%. The best degradation yield was shown when 3% NaCl was added, decomposing almost the entire PHB film. However, the degradation yield considerably decreased when more than 5% of NaCl was added, potentially because the high salt concentration burdened the cell and negatively affected the degradation potential.

In addition, different types of carbon and nitrogen sources were added to observe their effect on biodegradation and to improve the degradation yield of the cell. For the carbon sources, galactose, glycerol, lactose, glucose, fructose, sucrose, and xylose were added at 1% (*w/v*) concentration. The residual PHB films were compared to the control group cultured without carbon sources (Figure 2c). The results showed reduced degradation in the presence of additional carbon sources, except for xylose, which entirely degraded the PHB film. However, the degradation yield sharply decreased when glucose was added. This phenomenon seems to be related to carbon catabolite repression, which frequently occurs during PHB degradation, implying that the cell preferentially utilizes a favorable carbon source, thereby impeding the biodegradation of PHB [5,26,27]. In this case, the cell preferred to utilize glucose before PHB, resulting in the repression of PHB degradation, thus showing a degradation yield remarkably inferior to that of the control. Because xylose improved the biodegradation yield of *Microbulbifer* sp. SOL66, the optimal concentration, which could increase the degradation yield, was observed (Figure 2d). As a result, the degradation yield showed the highest degradation yield when 1% xylose was added, reaching 90% degradation yield over 3 d. However, the degradation yield decreased when more than 1.5% xylose was added.

In addition to the carbon sources, four types of nitrogen sources (ammonium nitrate [NH_4_NO_3_], ammonium sulfate [(NH_4_)_2_SO_4_], ammonium chloride [NH_4_Cl], and ammonium dihydrogen phosphate [(NH_4_)H₂PO_4_; 0.1%], were added to the culture medium of *Microbulbifer* sp. SOL66 (Figure 2e). The results showed a maximum degradation yield when ammonium sulfate was added, reaching more than 95%. Generally, nitrogen sources affect the synthesis and secretion of enzymes, and ammonium sulfate positively affects the cell in improving degradation activity [28]. In contrast, the degradation yield dropped to 70% when ammonium dihydrogen phosphate was added. It is assumed that ammonium dihydrogen phosphate impeded the biodegradation activity of the cell as cell aggregates formed after a few days of cultivation. To establish the optimal ammonium sulfate concentration for biodegradation, the culture medium was supplemented with up to 0.5% ammonium sulfate (Figure 2f). The best degradation yield was shown when 0.4% ammonium sulfate was added, reaching approximately 90% in 3 d.

### 3.3. Physical Properties of PHB after Biodegradation

**Figure 3:**
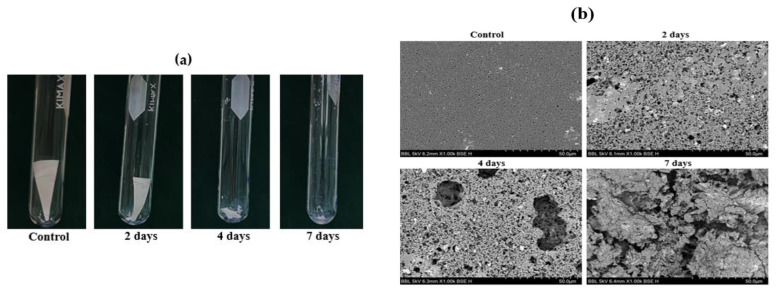
Degradation of poly(3-hydroxybutyrate) (PHB) and residual PHB film by *Microbulbifer* sp. SOL66. (**a**) The residual PHB films were collected on days 3, 5, and 7. (**b**) The surface of the degraded PHB film was observed using scanning electron microscopy (SEM) with magnified images.

Biodegradation of PHB progresses with changes in its physical properties, such as surface morphology, molecular weight, and functional groups. As PHB depolymerases work extracellularly from adhesion to the surface of the PHB, it is necessary to observe surface erosion and changes, which is one of the most noticeable changes throughout biodegradation [6,29,30]. Because *Microbulbifer* sp. SOL66 showed great biodegradation activity, it was cultivated with 100 mg of PHB pellets at 37 °C for 18 d to observe surface changes throughout biodegradation. The weight of the pellets did not decrease; however, surface changes in the pellets were detected after 18 d. The PHB pellets were collected, washed, and then coated with gold. The surface changes were observed using SEM and ultra-high resolution field emission scanning electron microscopy (Figure S1). Compared to the intact PHB pellet, which showed a smooth and even surface, the degraded PHB pellet showed erosion on a rough surface with pores. To obtain dramatically degraded samples, the PHB pellets were transformed into films using the solvent-cast method and cultivated with the cell. *Microbulbifer* sp. SOL66 cells were cultured in liquid MB medium with a PHB film at 37 °C for 7 d, and the residual PHB films were collected (Figure 3a). The amount of residual PHB films gradually decreased, resulting in the cleavage of the PHB film into several pieces after 4 d of cultivation. Finally, only a small amount of degraded PHB particles was collected after 7 d of cultivation. To obtain detailed images of the degraded films, the collected PHB films were washed with distilled water to remove cell residue and coated with gold to prevent edge effects and to obtain clear images using SEM. The samples were observed at 1000× magnification, which was suitable for observing surface changes (Figure 3b). Compared to the control, which showed a smooth surface, the degraded PHB films showed a rough and porous surface. It seemed that surface erosion was ongoing in the first 2 d, showing a partially rough surface, and after 4 d of cultivation, the whole surface became uneven, and large pores formed. On the final day, large cracks appeared on the surface, leaving the entire film destroyed.

**Figure 4:**
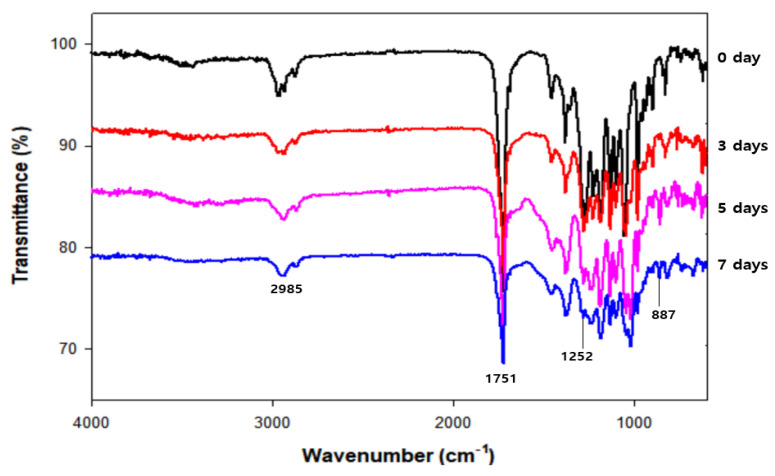
Change of physical properties of degraded poly(3-hydroxybutyrate) (PHB) analyzed by Fourier transform infrared spectroscopy (FT-IR) to observe some changes in functional groups after degradation.

In addition to the surface morphology, the functional groups in the degraded PHB film were analyzed using FTIR (Figure 4). Changes in several peaks were detected compared with the intact PHB film. For instance, the peak at 2985 cm^−1^ showed differences in the degraded PHB films, indicating that the C–H stretching bond had some changes throughout biodegradation. Likewise, the intensity of peaks at wavenumbers of 1252 cm^−1^ and 887 cm^−1^ considerably decreased during biodegradation, representing C–O stretching and C–H bending bonds, respectively [31]. In particular, the intensity of the peak at 1751 cm^−1^ showed a significant reduction, indicating that the C=O ester bond was affected throughout biodegradation. This phenomenon might be attributed to the cleavage of PHB, which is a polymer that is connected by the ester bonds of its monomers. From the FTIR data, it was observed that the PHB film was affected by *Microbulbifer* sp. SOL66 during cultivation.

Finally, the molecular weights (M_w_) of the degraded PHB films were analyzed using gel permeation chromatography (GPC). Because PHB is composed of long chains of monomers, it is broken down into smaller oligomers or monomers with various molecular weights during biodegradation [32]. For sample preparation, residual PHB films were collected, washed, and lyophilized. The samples were then dissolved in chloroform at 60 °C and re-precipitated with ice-cold water. The organic phase was collected and dissolved in chloroform. This procedure could remove impurities in the PHB film so that it could be easily dissolved in chloroform after re-precipitation. Finally, the samples were filtered through a syringe filter with a pore size of 0.2 μm and analyzed using GPC (Table 2).

As a result, the molecular weight of the PHB film decreased throughout biodegradation. The intact PHB film showed a molecular weight of 587 × 10^3^, followed by 319 × 10^3^, 220 × 10^3^, and 194 × 10^3^ as the biodegradation progressed. The polydispersity index (PDI), representing the distribution of molecular weights in a polymer, showed a PDI of 1.34 at first, which then increased to 5 during biodegradation. This phenomenon might be due to the enzymatic scission of PHB, resulting in a mixture of oligomers with various molecular weights, which broadened the distribution of the PHB molecular weights. Based on these changes accompanied by biodegradation, the enzyme excreted from *Microbulbifer* sp. SOL66 was shown to be effective for the biodegradation of PHB films.

### 3.4. Biodegradability of Other Bioplastics by Microbulbifer sp. SOL66

**Figure 5:**
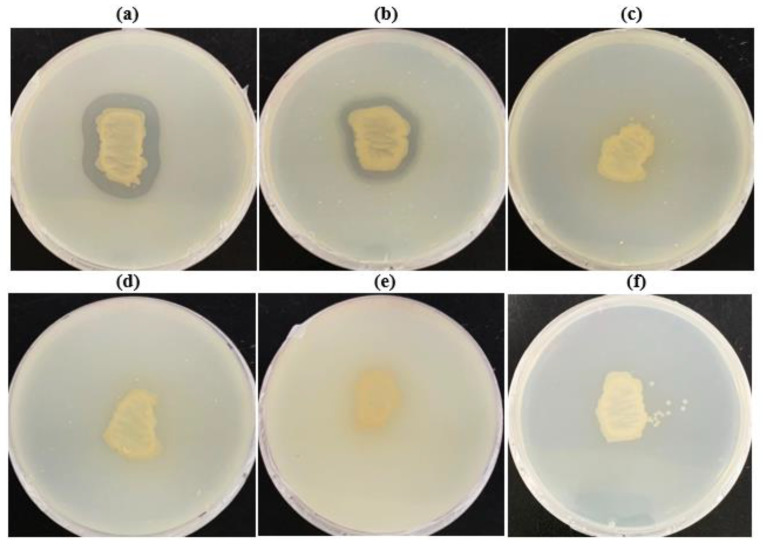
Biodegradation activity of the *Microbulbifer* sp. SOL66 cells were applied to other bioplastics. (**a**) Poly(3-hydroxybutyrate-co-4-hydroxybutyrate) [(P(3HB)-co-4HB)], (**b**) poly(3-hydroxybutyrate-*co*-3-hydroxyvalerate) [P(3HB-HV)], (**c**) polybutylene succinate (PBS), (**d**) polybutylene adipate terephthalate (PBAT), (**e**) polycaprolactone (PCL), (**f**) polylactic acid (PLA). The cell was cultured on the plates containing bioplastic emulsion, and the formation of the clear zone was observed.

Because *Microbulbifer* sp. SOL66 has a great biodegradation activity in PHB, biodegradability in other aliphatic bioplastics, such as PHB, was tested (Figure 5).

Copolymers, including P(60 mol% 3HB-*co*-40 mol% 4HB), P(88 mol%3HB-*co*-12 mol% HV), PBS, PBAT, PCL, and PLA, were used for the experiments. As a result, *Microbulbifer* sp. SOL66 could degrade both copolymers, and it formed a large clear zone on the P(3HB-*co*-4HB) plate in 3 d. To elucidate the utilization of 4-hydroxybutyrate (4HB) and 3-hydroxybutyrate (3HB) by the strain, metabolites in the culture medium were analyzed using GC-MS (data not shown). When the cells were cultured with PHB, succinic acid, formic acid, citric acid, and 3HB were produced as metabolites. In particular, 3HB, which is a monomer of PHB, is presented due to PHB cleavage during biodegradation. In contrast, when the cells were cultured with P(3HB-*co*-4HB), it produced 4HB, which is a monomer of the copolymer, succinic acid, and only a small amount of 3HB. The results indicated that *Microbulbifer* sp. SOL66 could utilize both 3HB and 4HB, resulting in the presence of each monomer in the culture medium. However, it seemed that the cell preferred to use 3HB before 4HB; thus, only a small amount of 3HB in the culture medium of the copolymer was present, leaving a large amount of 4HB (Figure S2).

**Figure 6:**
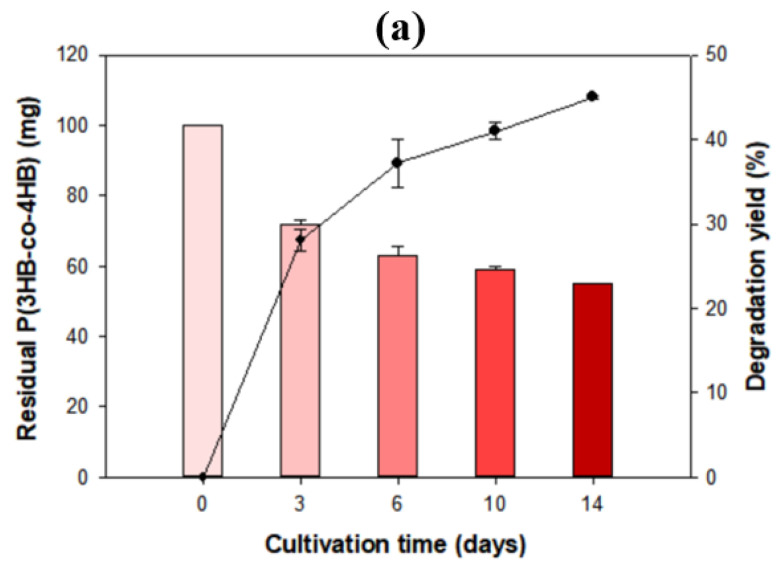

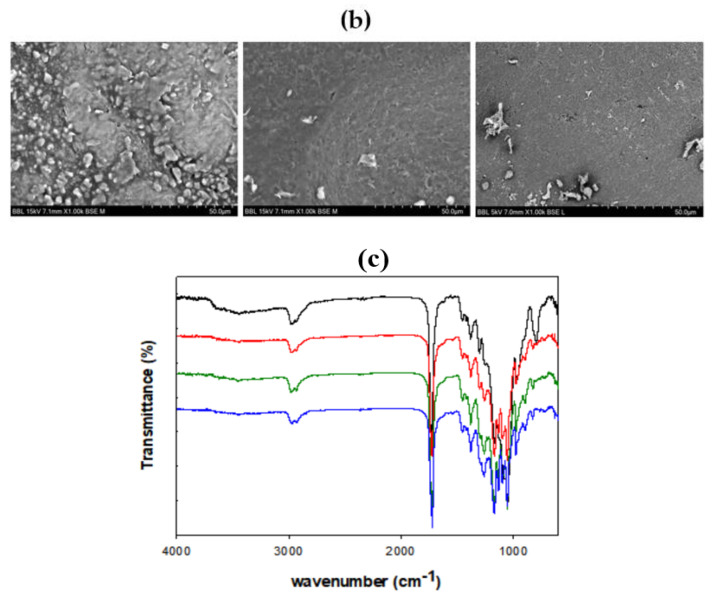
Biodegradability on the pellet of poly(3-hydroxybutyrate-*co*-4-hydroxybutyrate) [P(3HB-co-4HB)]. (**a**) *Microbulbifer* sp. SOL66 was cultured with P(3HB-co-4HB) pellets in a liquid marine broth (MB) medium, and the residual pellets were collected and weighed using an electronic scale. (**b**) The surface change of the degraded pellets was observed using scanning electron microscopy (SEM). The images are obtained with a sample of the control, 6 d, and 10 d, respectively. (**c**) Changes in Fourier transform infrared spectroscopy (FT-IR).

For detailed data, *Microbulbifer* sp. SOL66 was cultivated with the P(3HB-*co*-4HB) resin and further experiments were performed. Because the resin was a highly viscous substance, it was challenging to make a film of it. Thus, *Microbulbifer* sp. SOL66 was cultivated in liquid MB medium with 100 mg of P(3HB-*co*-4HB) resin. The residual resin was collected and washed, followed by lyophilization, and the weight loss was measured gravimetrically (Figure 6a). The results showed a significant decrease in weight in 14 d. During the first 3 d, a rapid decrease, representing approximately 30% of weight loss, and a gradual decrease, reaching approximately 45% of weight loss, was observed in 14 d. In addition to weight loss, the surface morphology was observed using SEM (Figure 6b). Compared to the intact PHB, the P(3HB-*co*-4HB) resin showed a significantly different surface owing to its high viscosity. The intact resin showed a smooth and glossy surface; however, after a few days of cultivation, it showed a rough surface with a small crack on it. Unlike the surface of the PHB, distinct differences were not detected on the surface of the P(3HB-*co*-4HB), which seemed to be due to its high viscosity, which prevented an attack by the microbial strains [33]. Changes in the functional groups were also analyzed (Figure 6c), with peaks appearing at 2983 cm^−1^ (C–H stretching), 1733 cm^−1^ (C=O stretching), 1167 cm^−1^ (C–O stretching), 1053 cm^−1^ (C–O stretching), and 801 cm^−1^ (C–H bending). In particular, the intensity of the peaks at 801 cm^−1^, 1053 cm^−1^, and 1167 cm^−1^ considerably decreased, compared to the Fourier transform infrared spectroscopy data of the PHB film, indicating that ester and aliphatic ether bonds were mainly affected by the biodegradation activity of the cell.

## 4. Conclusions

With the increasing demand for eco-friendly changes to petroleum-based plastics, bioplastics that are degradable by microorganisms are gaining attention worldwide. PHB is one of the most commercialized bioplastics, and its disposal has also been studied along with its use. We isolated several PHB-degrading bacteria using the systemic procedure from screening PHB-degrading bacteria we had established in a previous study to confirm their biodegradation activity. Because little is known about the biodegradation activity of *Microbulbifer* sp., one of the species was selected for further experiments. The degradation yield of *Microbulbifer* sp. SOL66 was confirmed by comparing the residual PHB (mg) to the intact PHB film, and the results showed that it reached approximately 98% degradation yield in 4 d, and 20 mg of the PHB film completely degraded within 7 d. In addition, there was a gradual increase in the clear zone on the PHB plates, confirming its biodegradation activity on PHB. To optimize the biodegradation of PHB, the temperature, salt concentration, and carbon and nitrogen source additions were investigated. The results showed that *Microbulbifer* sp. SOL66 exhibited the best degradation activity at 37 °C in the presence of 3% NaCl. In addition, supplementing 1% xylose and 0.4% ammonium sulfate resulted in the greatest degradation activity of the strain. In addition to the optimal conditions for biodegradation, the degraded PHB films were observed through changes in physical properties, such as surface morphology, functional groups, and molecular weights, compared to the intact PHB film. As the degradation progressed, the surface became rough with the appearance of large cracks, and some changes in functional groups, especially in ester bonds, were observed. Finally, decreasing molecular weight throughout biodegradation demonstrated that *Microbulbifer* sp. SOL66 exhibited high biodegradation activity. In addition, the biodegradability of P(3HB-*co*-4HB) was observed, and it showed approximately 45% weight loss in 14 d with some changes in physical properties, as mentioned above. 

According to our results presented above, we confirmed that *Microbulbifer* sp. SOL66 has a great biodegradation activity in PHB. With respect to our studies, the whole procedure from screening PHB-degrading bacteria to evaluating their biodegradation acitivity can broaden the database in the biodegradation field. Furthermore, by appling the *Microbulbifer* sp. SOL66 to degradation of other bioplastics than the PHB, it is expected to influence biodegradation studies regarding many other bioplastics, which will allow our lives to become familiar with using bioplastics that can be disposed of in an eco-friendly way. 

## Figures and Tables

**Table 1:** Strains used in this experiment.

Isolates	Similar Strain	Identity
SOL03	*Microbulbifer taiwanensis*	95.81%
SOL51	*Microbulbifer aestuariivivens*	94.18%
SOL55	*Microbulbifer salipaludis*	80.01%
SOL66	*Microbulbifer hydrolyticus*	95.15%
SOL84	*Microbulbifer elonatus*	97.01%

**Table 2:** Strains used in this experiment.

	Mw (×10^3^)	Mn (×10^3^)	Mw/Mn (PDI)
Control	587	438	1.34
3 days	319	57	5.56
5 days	220	40	5.53
7 days	194	35	5.59

## Data Availability

Not applicable. No new data were created or analyzed in this study. Data sharing is not applicable to this article.

## Supplementary Materials

The following are available online at https://www.mdpi.com/article/10.3390/polym13234257/s1, Figure S1: Surface change of the PHB pellet after 18 days cultivation, Figure S2: GC-MS data of the supernatant of culture medium of *Microbulbifer* sp. SOL66 with P(3HB-co-4HB).
