# Supplementary material for: Isolation of Microbulbifer sp. SOL66 with High Polyhydroxyalkanoate-Degrading Activity from the Marine Environment
Source: Polymers (Basel). 2021 Dec 4;13(23):4257. doi: 10.3390/polym13234257 (PMC8659741; doi:10.3390/polym13234257)

Supplementary Information

Isolation of *Microbulbifer* sp. SOL66 with High Polyhydroxyalkanoate-Degrading Activity from the Marine Environment

Sol Lee Park [1], Jang Yeon Cho [1], Soo Hyun Kim [1], Shashi Kant Bhatia [1,2], Ranjit Gurav [1], See-Hyoung Park [3], Kyungmoon Park [3] and Yung-Hun Yang [1,2,*]

1 Department of Biological Engineering, College of Engineering, Konkuk University, Seoul 05029, Korea; shckd20206@naver.com (S.L.P.); whwkddus1123@gmail.com (J.Y.C.); gsm06136@naver.com (S.H.K.); shashibiotechhpu@gmail.com (S.K.B.); rnjtgurav@gmail.com (R.G.)
2 Institute for Ubiquitous Information Technology and Applications, Konkuk University, Seoul 05029, Korea
3 Department of Biological and Chemical Engineering, Hongik University, Sejong 30016, Korea; shpark74@hongik.ac.kr (S.-H.P.); pkm2510@hongik.ac.kr (K.P.)
* Correspondence: seokor@konkuk.ac.kr; Tel.: +82-2-450-3936

Figure S1: Surface change of the PHB pellet after 18 days cultivation.

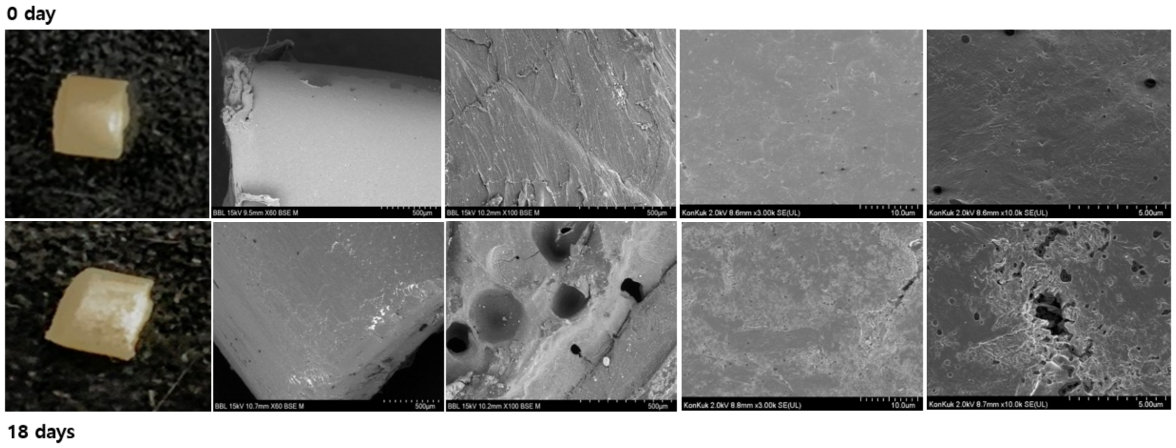

Figure S2: GC-MS data of the supernatant of culture medium of *Microbulbifer* sp. SOL66 with P(3HB-*co*-4HB).

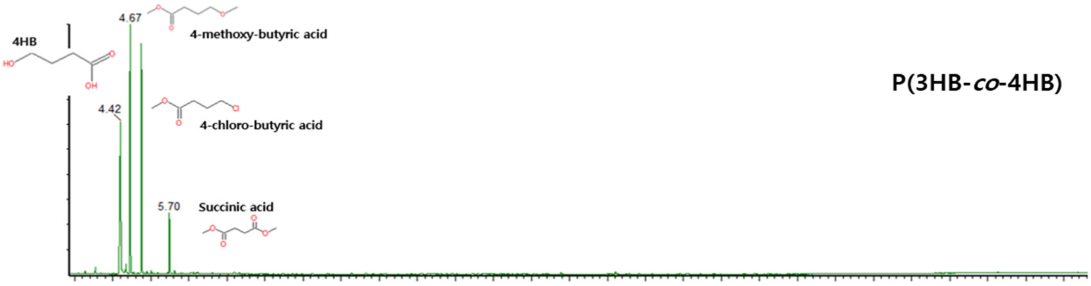

Supplement: Supplementary file 1 [file polymers-13-04257-s001.zip › polymers-1468545-supplementary.pdf]
